# Blood Lead Level and Measured Glomerular Filtration Rate in Children with Chronic Kidney Disease

**DOI:** 10.1289/ehp.1205164

**Published:** 2013-05-21

**Authors:** Jeffrey J. Fadrowski, Alison G. Abraham, Ana Navas-Acien, Eliseo Guallar, Virginia M. Weaver, Susan L. Furth

**Affiliations:** 1 Department of Pediatrics, Johns Hopkins University School of Medicine, Baltimore, Maryland, USA; 2 Welch Center for Prevention, Epidemiology and Clinical Research, Johns Hopkins University School of Medicine and Bloomberg School Public Health, Baltimore, Maryland, USA; 3 Department of Epidemiology, and; 4 Department of Environmental Health Sciences, Johns Hopkins University Bloomberg School of Public Health, Baltimore, Maryland, USA; 5 Department of Medicine, Johns Hopkins University School of Medicine, Baltimore, Maryland, USA; 6 Department of Epidemiology and Population Genetics, National Center for Cardiovascular Research, Madrid, Spain; 7 Department of Pediatrics, University of Pennsylvania School of Medicine, Philadelphia, Pennsylvania, USA

**Keywords:** children, chronic kidney disease, kidney, lead, nephrotoxicity, pediatric

## Abstract

Background: The role of environmental exposure to lead as a risk factor for chronic kidney disease (CKD) and its progression remains controversial, and most studies have been limited by a lack of direct glomerular filtration rate (GFR) measurement.

Objective: We evaluated the association between lead exposure and GFR in children with CKD.

Methods: In this cross-sectional study, we examined the association between blood lead levels (BLLs) and GFR measured by the plasma disappearance of iohexol among 391 participants in the Chronic Kidney Disease in Children (CKiD) prospective cohort study.

Results: Median BLL and GFR were 1.2 µg/dL and 44.4 mL/min per 1.73 m^2^, respectively. The average percent change in GFR for each 1-µg/dL increase in BLL was –2.1 (95% CI: –6.0, 1.8). In analyses stratified by CKD diagnosis, the association between BLL and GFR was stronger among children with glomerular disease underlying CKD; in this group, each 1-µg/dL increase in BLL was associated with a –12.1 (95% CI: –22.2, –1.9) percent change in GFR. In analyses stratified by anemia status, each 1-µg/dL increase in BLL among those with and without anemia was associated with a –0.3 (95% CI: –7.2, 6.6) and –4.6 (95% CI: –8.9, –0.3) percent change in GFR, respectively.

Conclusions: There was no significant association between BLL and directly measured GFR in this relatively large cohort of children with CKD, although associations were observed in some subgroups. Longitudinal analyses are needed to examine the temporal relationship between lead and GFR decline, and to further examine the impact of underlying cause of CKD and anemia/hemoglobin status among patients with CKD.

## Introduction

Although lead levels have decreased in the general population over the past few decades, lead remains a widespread environmental toxicant [Centers for Disease Control and Prevention (CDC) 2009]. Lead is associated with numerous adverse health effects, including kidney disease [Agency for Toxic Substances and Disease Registry (ATSDR) 2007]. High chronic lead exposure (blood levels > 70–80 µg/dL) is an established cause of nephropathy in adults and children (Ekong et al. 2006; Inglis et al. 1978; Khalil-Manesh et al. 1992; Steenland et al. 1992; Wedeen et al. 1979). At lead levels representative of current environmental exposure (blood levels < 10 µg/dL), several cross-sectional and a few prospective studies have reported an association with kidney dysfunction or progression of chronic kidney disease (CKD) (Åkesson et al. 2005; Ekong et al. 2006; Fadrowski et al. 2010; Kim et al. 1996; Lin et al. 2003, 2006; Muntner et al. 2003, 2005; Navas-Acien et al. 2009; Payton et al. 1994; Staessen et al. 1992; Tsaih et al. 2004; Yu et al. 2004). However, data in children are scarce and less consistent than in adults (de Burbure et al. 2006; Fadrowski et al. 2010; Moel and Sachs 1992; Staessen et al. 2001).

Furthermore, most studies of the association between lead and CKD evaluated glomerular filtration rate (GFR) using estimating equations based on serum creatinine or cystatin C (Spector et al. 2011). These equations have limited precision and accuracy compared with formal measurement of GFR (Fadrowski et al. 2011; Poggio et al. 2005; Rule et al. 2004; Schwartz et al. 2009; Staples et al. 2010; Stevens et al. 2007), and lack of formal measurement of GFR is commonly listed as a limitation in studies examining the impact of lead on the kidney.

The ongoing National Institutes of Health–sponsored Chronic Kidney Disease in Children (CKiD) prospective cohort study has a primary aim of characterizing traditional and nontraditional risk factors for CKD progression (Furth et al. 2006). CKiD directly measures GFR via the plasma disappearance of iohexol, providing a unique opportunity to examine the impact of environmental exposures using measured GFR (Schwartz et al. 2006). Therefore, we conducted an ancillary study within CKiD to examine the association between blood lead levels and iohexol GFR in children and adolescents 1–19 years of age.

## Methods

*Study setting, design, and population*. The CKiD study is a prospective cohort study to identify risk factors for CKD progression (Copelovitch et al. 2011; Furth et al. 2006). As of 2011, 586 children 1–16 years of age with CKD of various etiologies and an estimated GFR of 30–90 mL/min per 1.73 m^2^ by the Schwartz formula (Schwartz et al. 1987, 1976) have been enrolled from 48 clinical sites in the United States and Canada. The protocol for this study and the informed consent procedures were included in the main protocol for the CKiD study and approved by the institutional review boards at each participating center.

Enrollment of the CKiD cohort occurred over an approximately 2-year period. The present ancillary study collected whole blood aliquots for lead analysis in study participants starting several months after the cohort began year 2 study visits, and thus a portion of the cohort is missing year 2 lead values. Of 500 children completing year 2 visits, 382 had lead levels available (collected between January 2007 and December 2009). Of 211 children completing year 4 visits, 201 had lead levels available (collected between January 2008 and December 2009).

For the present cross-sectional analysis, we included all participants with blood lead levels from years 2 and/or 4 of the study (*n* = 456, contributing 583 lead measurements). We excluded participants who were missing data on Hispanic ethnicity (*n* = 7), body mass index (BMI) (*n* = 21), proteinuria (*n* = 24), income relative to the poverty level (*n* = 36), and hemoglobin (*n* = 10), leading to a final sample size of 391 participants contributing 485 lead measurements.

*Analysis of blood lead*. Lead and cadmium levels in whole blood were measured by high resolution inductively coupled plasma mass spectrometry at the University of California, Santa Cruz, Environmental Toxicology Laboratory (Smith DR). Samples were analyzed on an Element XR inductively coupled plasma mass spectrometer (Thermo Scientific, West Palm Beach, FL, USA) using standardized protocols including confirmation that storage materials were not contaminated with background lead. No samples were below the analytical limit of detection (< 0.1 µg/dL). Accuracy was assessed using National Institute of Standards and Technology (NIST; Gaithersburg, MD, USA) standard reference materials (SRMs). Analyses using SRMs reflecting blood lead levels of 1.6 µg/dL and 25.3 µg/dL had percent relative standard deviations (%RSDs) of 4.6 and 5.5, respectively. We assessed reproducibility by *a*) analyzing replicate samples at intervals throughout the same analytic run, *b*) analyzing samples in triplicate in the same run, and *c*) analyzing replicate samples in separate runs. Percent RSD for all reproducibility determinations was < 2.5%.

*GFR*. GFR was measured at years 2 and 4 of the CKiD study based on plasma disappearance curves of iohexol (Ominipague; GE Healthcare, Princeton, NJ, USA). Iohexol (5 mL) was administered intravenously and blood samples were obtained at four time points at 10, 30, 120, and 300 min after infusion based on pilot data (Schwartz et al. 2006). Of the 485 observations used herein, 30 (6.2%) did not have successful iohexol GFRs. In these cases, GFR was estimated by a CKiD-derived GFR estimating equation (Schwartz et al. 2009):

eGFR = 40.7 × (height/serum creatinine)^0.64^ × (30/blood urea nitrogen)^0.202^,

with height in meters, and serum creatinine and blood urea nitrogen in milligrams per deciliter. GFR estimated by the “bedside CKiD” equation [eGFR = 41.3(height/serum creatinine)] (Schwartz et al. 2009) was also examined in a sensitivity analysis. Serum creatinine and blood urea nitrogen were analyzed at the CKiD central laboratory on an Advia 2400 (Siemens Diagnostics, Tarrytown, NY, USA). It has been previously shown that the Siemens Bayer Advia creatinine measurement closely agrees with the high-performance liquid chromatography method traceable to reference isotope dilution mass spectroscopy developed by the NIST (Schwartz et al. 2006).

*Other variables*. BMI was calculated as weight in kilograms divided by height in meters squared. BMI percentiles were calculated based on the CDC’s BMI-for-age sex-specific growth charts, and participants were categorized as obese if their BMI was at the 95th percentile or higher (CDC 2012a). The diagnoses of CKD were reviewed by the CKiD Steering Committee and categorized as either glomerular or nonglomerular. Glomerular diagnoses include chronic glomerulonephritis, congenital nephrotic syndrome, diffuse mesangial sclerosis (Denys–Drash syndrome), diabetic nephropathy, familial nephritis, focal segmental glomerulosclerosis, hemolytic uremic syndrome, Henoch–Schönlein nephritis, idiopathic crescentic glomerulonephritis, IgA nephropathy, membranoproliferative glomerulonephritis types I and II, membranous nephropathy, sickle cell nephropathy, and systemic immunologic disease including systemic lupus erythematosus. Nonglomerular diagnoses included aplastic, hypoplastic, and dysplastic kidneys, cystinosis, medullary cystic disease/juvenile nephronophthisis, obstructive uropathy, oxalosis, autosomal dominant and recessive polycystic kidney disease, pyelonephritis/interstitial nephritis, reflux nephropathy, renal infarct, syndrome of agenesis of abdominal musculature, and Wilm’s tumor. A CKD diagnosis not included by one of the above was reviewed by the steering committee and, if necessary, discussed with the clinical site to be certain that it was properly categorized as glomerular or nonglomerular. Proteinuria was categorized by calculated first morning urine protein to creatinine ratio (UPC): none, UPC ≤ 0.2; significant, UPC > 0.2 to < 2.0; and nephrotic, UPC ≥ 2.0. Poverty was defined based on participant household size and income using 2009 U.S. Federal Poverty Guidelines (U.S. Department of Health and Human Services 2009). Anemia was defined as hemoglobin level less than the 5th percentile for age and sex. For secondary analyses, an “anemia status” variable was categorized as anemic participants, not treated with an erythropoiesis stimulating agent (ESA) (for example, erythropoietin); participants without anemia and not treated with an ESA; and participants treated with an ESA, with or without anemia.

*Statistical analysis*. Median and interquartile ranges (25th–75th percentiles) for blood lead levels and GFR were calculated for the entire study population. *p*-Values were determined using the median command in Stata which performs a nonparametric K-sample test on the equality of the medians and provides a Pearson chi-square test statistic. Linear regression was used to estimate associations between blood lead levels and GFR. Non-independence between measures from the same person (*n* = 94 with two measurements) was accounted for using robust standard errors. As a sensitivity analysis, models were rerun using linear mixed effect models in SAS and showed similar results (data not shown). Lead exposure, the explanatory variable in the linear regression model, was modeled as an untransformed continuous variable or as a natural log (ln)–transformed continuous variable. Because inferences based on ln-transformed lead were comparable (data not shown), results are reported for lead modeled as an untransformed variable for ease of interpretation. GFR was ln-transformed because it was not normally distributed. Continuous covariates (age, BMI *z*-score, and urine protein:creatinine ratio in the main analysis, and ln-transformed blood cadmium and ln-transformed hemoglobin in secondary analyses) were centered at the median.

Linear regression models were fitted with increasing degrees of adjustment. First we adjusted for age (continuous), sex, race (black, white, or other), Hispanic ethnicity, BMI *z*-score (continuous), and poverty (yes/no). Second, the model was further adjusted for CKD diagnosis (glomerular or nonglomerular) and urine protein to creatinine ratio (continuous). Finally, the model was further adjusted for ln-transformed blood cadmium level (continuous). The estimated percent change in GFR associated with a 1-µg/dL increase in blood lead was approximated by 100 × β, where β is the coefficient for blood lead from the linear regression model of ln-GFR. For ease of interpretation, the main result is also reported for GFR as an untransformed dependent variable (with units of milliliters per minute per 1.73 m^2^). To accomplish this, the beta and intercept from the original ln-tranformed GFR model are exponentiated, and thus the estimate corresponds to the change in GFR in milliliters per minute per 1.73 m^2^ for an individual who is female, white, not Hispanic, not impoverished, not diagnosed with glomerular CKD, and of median age, BMI *z*-score, urine protein to creatinine ratio, and ln-transformed blood cadmium level (the reference category of each variable). Hypertension (yes/no) and blood pressure variables (systolic/diastolic blood pressure *z*-scores/percentiles) were also evaluated as covariates but were not included in the fully adjusted final model because they did not influence the magnitude of the association between lead and GFR (data not shown) (National High Blood Pressure Education Program Working Group on High Blood Pressure in Children and Adolescents 2004). Analyses were also restricted to participants without missing iohexol GFR (455 measurements) with similar results (data not shown).

To evaluate possible nonlinear associations between blood lead level and ln-GFR, a linear–linear spline regression analysis in fully adjusted models was examined with the cut point (1 µg/dL) selected post hoc to maximize the differences in the slopes of the linear segments above and below the cut point.

In secondary analyses, models were stratified by the participant characteristics presented in Table 1, except for anemia. For the stratified analysis, proteinuria was defined as a urine protein to creatinine ratio > 0.2. *p*-Values for interaction are the Wald *p*-values for cross-product (interaction) terms between lead and each participant characteristic. In addition, we estimated associations stratified by anemia status, with and without hemoglobin adjustment.

**Table 1:** Blood lead levels and GFR by participant characteristic.^*a*^

Characteristic	No. (%) of participants	Blood lead level(µg/dL)	*p*-Value	GFR(mL/min/1.73m^2^)	*p*-Value
Total	391	1.2 (0.9, 1.8)	—	44.4 (33.7, 57.9)	—
Sex
Female	154 (39)	1.1 (0.8, 1.5)	0.02	45.3 (34.7, 56.6)	0.51
Male	237 (61)	1.3 (1.0, 1.9)		43.7 (32.8, 58.8)	
Age (years)
0–5	50 (13)	1.7 (1.1, 2.8)	<0.001	44.4 (36.9, 58.3)	0.72
6–11	149 (38)	1.3 (1.0, 1.9)		44.9 (35.5, 56.7)	
12–19	192 (49)	1.1 (0.8, 1.5)		43.8 (32.1, 59.0)	
Race
Black	59 (15)	1.4 (1.0, 2.1)	0.02	47.3 (37.0, 68.4)	0.34
White	270 (69)	1.1 (0.8, 1.7)		43.5 (32.7, 55.6)	
Other	62 (16)	1.2 (1.0, 1.6)		47.6 (36.3, 63.1)	
Hispanic
Yes	51 (13)	1.1 (0.8, 1.6)	0.05	39.1 (32.7, 50.7)	0.01
No	340 (87)	1.2 (0.9, 1.8)		45.8 (34.4, 59.2)	
Obese^*b*^
Yes	57 (15)	1.1 (0.8, 1.6)	0.20	44.5 (32.6, 64.5)	0.65
No	334 (85)	1.2 (0.9, 1.8)		44.2 (34.1, 57.5)	
Poverty^*c*^
Yes	82 (21)	1.4 (1.0, 2.0)	0.01	43.7 (32.7, 65.2)	0.47
No	309 (79)	1.1 (0.8, 1.7)		44.6 (34.1, 57.5)	
CKD diagnosis^*d*^
Glomerular	73 (19)	0.9 (0.6, 1.2)	<0.001	48.3 (32.0, 65.6)	0.35
Nonglomerular	318 (81)	1.3 (1.0, 1.8)		44.1 (34.1, 56.6)	
Proteinuria^*e*^
None	114 (29)	1.1 (0.9, 1.8)	0.23	55.7 (44.0, 68.1)	<0.001
Significant	233 (60)	1.3 (0.9, 1.8)		42.7 (32.8, 53.5)	
Nephrotic	44 (11)	1.1 (0.9, 1.5)		31.8 (22.8, 41.7)	
Blood cadmium tertile(µg/L)
1 (≤0.097)	128 (33)	1.1 (0.8, 1.6)	0.27	45.3 (35.5, 59.3)	0.46
2 (0.097–≤0.16)	134 (34)	1.3 (0.9, 1.8)		43.6 (34.0, 55.1)	
3 (>0.16)	129 (33)	1.2 (1.0, 1.7)		44.7 (32.6, 61.7)	
Anemia^*f*^
Yes, untreated	107 (28)	1.2 (0.9, 1.9)	0.01	38.4 (28.3, 47.3)	<0.001
No, untreated	224 (57)	1.3 (1.0, 1.8)		51.8 (43.1, 65.4)	
ESA-treated	60 (15)	0.9 (0.7, 1.4)		30.6 (22.8, 36.8)	
^***a***^Characteristics from first (year 2) study visit only are presented for participants contributing data from more than one study visit (*n*=94). Data are given as median (interquartile range) unless otherwise indicated. ^***b***^Obesity was defined as BMI (weight in kilograms divided by height in meters squared) at or above the 95th percentile. ^***c***^Poverty definition based on 2009 U.S. Federal Poverty Guidelines (U.S. Department of Health and Human Services 2009) incorporating household income and family size. ^***d***^See “Methods” for complete listing of glomerular and nonglomerular diagnoses. ^***e***^Protein/­creatinine ratio >0.2 and <2.0; nephrotic defined as ratio ≥2.0. ^***f***^Hemoglobin level <5th percentile for age/sex; ESA-treated category includes participants with and without anemia.					

All statistical analyses were two-sided. The threshold for statistical significance for all analyses was set to 0.05. Data analyses were performed using Stata versions 11.0 and 12.0 (StataCorp, College Station, TX, USA) and SAS version 9.1 (SAS Institute Inc., Cary, NC, USA) statistical software.

## Results

The median blood lead level was 1.2 µg/dL (range, 0.2–6.2 µg/dL), and the median GFR was 44.4 (range, 11.9–156.4) mL/min per 1.73 m^2^ (Table 1). Blood lead levels were higher among males, younger children, black children, children living in poverty, children with nonglomerular causes of CKD, and children who were not treated with an ESA. GFR was lower among Hispanic children, children with proteinuria, and children with anemia or treated with an ESA.

In linear regression analysis, each 1-µg/dL increase in blood lead level was associated with an average percent change in GFR of –2.1 (95% CI: –6.0, 1.8; *p* = 0.29) after adjustment (Table 2, model 3). This corresponds to an average difference in GFR of –0.9 mL/min (95% CI: –2.6, 0.8 mL/min) per 1.73 m^2^. In analysis using a linear spline, each 1-µg/dL increase in blood lead level > 1 µg/dL was associated with a percent change in GFR of –3.8 (95% CI: –8.1, 0.4; *p* = 0.08); the corresponding estimate for a lead level < 1 µg/dL was 15.9 (95% CI: –9.7, 41.6; *p* = 0.22). In analyses estimating GFR by the bedside CKiD GFR estimating equation instead of using iohexol GFR, each 1-µg/dL increase in blood lead level was associated with a percent change in GFR of –2.5 (95% CI: –6.5, 1.6; *p* = 0.23).

**Table 2:** Estimated percent change (95% CI) in GFR per µg/dL increase in blood lead level.^*a*^

	Percent change per1-µg/dLincrease (95% CI)	*p-*Value
Unadjusted	–0.7 (–4.9, 3.5)	0.75
Model 1^*b*^	–1.6 (–5.8, 2.6)	0.44
Model 2^*c*^	–2.0 (–5.9, 1.9)	0.31
Model 3^*d*^	–2.1 (–6.0, 1.8)	0.29
^***a***^*n*=485 for all models; linear regression of lead as a continuous variable. ^***b***^Model 1 is adjusted for age, sex, race, Hispanic ethnicity, BMI *z*-score, and poverty. ^***c***^Model 2 is model 1 additionally adjusted for CKD diagnosis and urine protein:creatinine ratio. ^***d***^Model 3 is model 2 additionally adjusted for ln-­transformed blood cadmium level.		

Analyses stratified by sex, age, race, Hispanic ethnicity, obesity, poverty, and proteinuria subgroups showed associations similar to that found in the overall study population (Table 3). The association between blood lead level increase and change in GFR was significant among children in the highest cadmium tertile [percent change in GFR of –7.6 (95% CI: –13.6, –1.5) for the highest cadmium tertile, compared with 3.2 (95% CI: –3.7, 10.1) for the lowest]. However, interaction by blood cadmium level was not significant (*p* = 0.10).

**Table 3:** Estimated percent change in GFR (95% CI) per 1-µg/dL increase in blood lead level stratified by participant characteristic.^*a*^

Characteristic	Percent change per1-µg/dL increase (95% CI)^*b*^	*p-*Value for interaction^*c*^
Total	–2.1 (–6.0, 1.8)	NA
Sex
Male	–2.1 (–6.7, 2.6)	
Female	–1.5 (–8.6, 5.6)	0.69
Age (years)
0–5	–3.2 (–11.4, 5.1)	
6–11	–1.1 (–8.0, 5.7)	
12–19	–2.3 (–9.2, 4.6)	0.77
Race
Black	–1.9 (–11.9, 8.0)	
White	–2.1 (–6.6, 2.5)	
Other	1.3 (–12.2, 14.9)	0.63
Hispanic
Yes	–5.4 (–15.1, 4.3)	
No	–1.2 (–5.4, 3.0)	0.07
Obesity
Yes	–1.5 (–8.9, 5.8)	
No	–2.7 (–7.0, 1.6)	0.62
Poverty
Yes	–2.9 (–10.8, 5.0)	
No	–1.5 (–6.1, 3.1)	0.24
CKD diagnosis
Glomerular	–12.1 (–22.2, –1.9)	
Nonglomerular	–0.7 (–4.8, 3.4)	0.03
Proteinuria^*d*^
Yes	1.2 (–3.3, 5.7)	
No	–4.7 (–11.2, 1.8)	0.53
Blood cadmium tertile (µg/L)
1 (≤0.097)	3.2 (–3.7, 10.1)	
2 (0.097–≤0.16)	1.6 (–5.3, 8.5)	
3 (>0.16)	–7.6 (–13.6, –1.5)	0.10
NA, not applicable. ^***a***^*n*=485; linear regression of lead as continuous variable. ^***b***^Each stratified model adjusted for all other characteristics in table. Age, BMI *z*-score, urine protein:creatinine ratio, and ln-transformed blood cadmium modeled as continuous variables, centered at the median. ^***c***^Interaction tests based on the Wald test. ^***d***^Urine protein:creatinine ratio >0.2.		

In analyses stratified by CKD diagnosis, each 1-µg/dL increase in blood lead level was associated with a percent change in GFR of –12.1 (95% CI: –22.2, –1.9; *p* = 0.02) and –0.7 (95% CI: –4.8, 3.4; *p* = 0.74) in those with glomerular and nonglomerular CKD diagnoses, respectively (*p* for interaction by CKD diagnosis = 0.03). The geometric means for blood lead level and GFR adjusted for age, sex and race, by glomerular and nonglomerular diagnosis category, were 1.0 and 1.3 µg/dL (*p-*value for difference in means < 0.001), and 45.6 and 43.0 mL/min per 1.73 m^2^ (*p* = 0.33), respectively. The mean urine protein to creatinine ratios were 1.7 and 0.9 in children with glomerular and nonglomerular causes of CKD (*p* < 0.001). Final models were adjusted for proteinuria to exclude proteinuria as an explanatory factor for these findings. In addition, fully adjusted models stratified by proteinuria status showed no evidence of a difference in the association between lead and GFR based on the presence or absence of proteinuria (*p* = 0.53 for interaction) (data not shown). Among children with glomerular causes of CKD, 20% were hypertensive versus 13% of those with nonglomerular causes (*p* = 0.1). Sensitivity analyses including hypertension (*n* = 467) or anemia status (*n* = 485) in the final stratified model revealed similar results (data not shown).

Among all participants, median (interquartile range) hemoglobin level was 12.6 g/dL (11.7–13.6 g/dL). The mean, 5th, and 95th percentiles for hemoglobin were 12.6, 10.2, and 15.2 g/dL, respectively. The Spearman correlation coefficient between lead and hemoglobin was 0.12 (*p* = 0.008). Inclusion of ln-transformed hemoglobin in the fully adjusted model (corresponding to model 3 in Table 2, which does not include hemoglobin adjustment) was associated with a percent change in GFR of –3.9 (95% CI: –7.6, –0.3; *p* = 0.04) for every 1-µg/dL increase in blood lead level. In analyses stratified by anemia status (Table 4), the association between blood lead level and percent decrease in GFR was statistically significant among those who were not anemic (and not treated with an ESA) compared with those who were anemic or treated with an ESA. Hemoglobin adjustment did not affect these results (Table 4).

**Table 4:** Estimated percent change in GFR per 1‑µg/dL increase in blood lead level (95% CI) stratified by anemia ­status,^*a*^ without and with hemoglobin adjustment.^*b*^

	Untreated, anemic	Untreated, notanemic	Treated with ESA	*p*-Value for interaction^*c*^
No hemoglobin adjustment	–0.3 (–7.2, 6.6)	–4.6 (–8.9, –0.3)	–1.1 (–11.9, 9.7)	0.49
Hemoglobin adjusted^*d*^	–0.5 (–7.5, 6.5)	–5.1 (–9.2, –0.9)	–2.3 (–13.8, 9.2)	0.50
^***a***^Hemoglobin level <5th percentile for age/sex. ^***b***^Model adjusted for age, sex, race, Hispanic ethnicity, BMI *z*-score, poverty, CKD diagnosis, urine protein:creatinine ratio, and ln-transformed blood cadmium level. ^***c***^Interaction tests based on the Wald test. ^***d***^Model additionally adjusted for ln-transformed hemoglobin.				

## Discussion

In a large cohort of children with CKD and a median blood lead level of 1.2 µg/dL, higher blood lead level was not associated with lower measured GFR after adjustment for factors known to affect blood lead levels and/or GFR. For every 1-µg/dL increase in blood lead, the estimated percent change in GFR was –2.1 (95% CI: –6.0, 1.8). In analyses stratified by CKD diagnosis, the association between blood lead level and GFR was stronger among children with glomerular disease underlying CKD; in this group, each 1-µg/dL increase in blood lead was associated with a –12.1 (95% CI: –22.2, –1.9) percent change in GFR. In analyses stratified by anemia status, the association was stronger among participants who were not anemic and not being treated for anemia; each 1-µg/dL increase in blood lead was associated with a –4.6 (95% CI: –8.9, –0.3) percent change in GFR.

Blood lead levels in the CKiD cohort are similar to those measured around the same time period in a nationally representative sample of similarly aged children participating in the 2007–2008 National Health and Nutrition Examination Survey (NHANES) and thus representative of current levels of exposure from the environment (National Center for Health Statistics 2012). Mean blood lead levels in 2007–2008 NHANES were 1.5, 1.0, and 0.8 µg/dL in children 1–5, 6–11, and 12–19 years of age, respectively (CDC 2012b). Exposure to lead has decreased substantially in the United States over the past few decades, primarily owing to public health measures including the government-mandated ban of residential lead-based paint in 1978 and phaseout of leaded gasoline in the 1970s and 1980s (ATSDR 2007). Despite being born after the elimination of many common industrial uses of lead, the CKiD cohort and children in recent NHANES surveys indicate that lead exposure is ongoing, because most children in the U.S. population still have detectable blood levels. Current exposure sources include diet, industrial sources, decaying lead paint, soil contaminated with lead paint or through the use of leaded gasoline, tobacco smoke, folk remedies, glazed pottery, and drinking water in some urban areas (Apostolou et al. 2011; ATSDR 2007; Clayton et al. 1999; Lanphear et al. 1998; Levin et al. 2008; Lin et al. 2004). It is also known that certain populations continue to experience higher lead exposure, particularly inner-city children and adults living in areas of low socioeconomic status. Indeed, children living in impoverished households in the CKiD study had higher blood lead levels.

Previous studies examining the association between lead and kidney function in children and adults with and without kidney disease have reported conflicting associations (Ekong et al. 2006; Evans and Elinder 2011). A study of 769 adolescent NHANES participants with a median blood lead level of 1.5 µg/dL and a median creatinine-estimated GFR of 108.8 mL/min per 1.73 m^2^ found a decrease in creatinine-estimated GFR per doubling of blood lead of –1.0 (95% CI: –2.9, 0.9) mL/min per 1.73 m^2^ (Fadrowski et al. 2010) In the same cohort, the difference in cystatin C–estimated GFR per doubling of blood lead level was –2.9 mL/min (95% CI: –5.0, –0.7 mL/min) per 1.73 m^2^. A positive correlation between blood lead levels and serum cystatin C was reported for a study of 200 European adolescents (Staessen et al. 2001), but blood lead levels were negatively correlated with serum creatinine and cystatin C in a study of > 800 children in Europe (de Burbure et al. 2006). Low-level environmental lead exposure has been associated with decreased kidney function in several cross-sectional and a few prospective studies in adults (Åkesson et al. 2005; Ekong et al. 2006; Kim et al. 1996; Lin et al. 2006; Muntner et al. 2003, 2005; Payton et al. 1994; Staessen et al. 1992; Tsaih et al. 2004). In a prospective study of 121 adults with CKD and a mean baseline blood lead level of 4.2 µg/dL, Yu et al. (2004) estimated an annual decline of 1 mL/min per 1.73 m^2^ per 1-µg/dL increment in baseline lead level. In a randomized clinical trial of 64 patients with CKD, an increase in GFR of 2.1 mL/min per 1.73 m^2^ was estimated for the group receiving chelation therapy compared with a 6.0 mL/min per 1.73 m^2^ GFR decline among controls during a 27-month follow-up period (*p* < 0.001) (Lin et al. 2003; Lin-Tan et al. 2007).

In our study population, the negative association between blood lead and GFR was stronger in children with CKD attributed to glomerular causes. Most cross-sectional studies examining the impact of lead on the kidney have not examined a population with known CKD, so examining a differential impact by CKD diagnosis has not been possible. The few previous studies of CKD patients have adjusted estimated associations for CKD diagnosis, including a “chronic glomerulonephritis” category, and have not reported results of stratified analyses (Lin et al. 2001, 2003, 2006; Yu et al. 2004). Glomerular diagnoses have been associated with increased proteinuria regardless of GFR in children with CKD (Wong et al. 2009), and proteinuria is an established risk factor for lower GFR and GFR decline in both children and adults [Ardissino et al. 2004; GISEN Group (Gruppo Italiano di Studi Epidemiologici in Nefrologia) 1997; Litwin 2004; Peterson et al. 1995; Wingen et al. 1997; Wong et al. 2006; Wuhl et al. 2004]. Our associations, however, were adjusted for proteinuria, and effect modification was not statistically significant. Furthermore, associations were unchanged when we adjusted for hypertensive or anemia status. Patients with glomerular disease might have more severe CKD or exposures to medications such as corticosteroids, and thus more advanced bone disease, which might lead to higher blood levels due to increased bone turnover. However, mean age-, sex-, and race-adjusted blood lead levels were actually lower among children with glomerular compared with nonglomerular causes of CKD (1.0 vs. 1.3 µg/dL, respectively). Mean GFR was also higher in children with glomerular compared with nonglomerular CKD (45.6 vs. 43.0 mL/min per 1.73 m^2^, respectively).

Adding hemoglobin to the fully adjusted model strengthened the negative association between blood lead levels and GFR. In analyses stratified by anemia status, the association was stronger and reached statistical significance among those participants who were not anemic. Median lead levels were similar between anemic and nonanemic participants. Median GFR was higher among those without anemia, as would be expected given the well described relationship between GFR and hemoglobin among those with CKD (Fadrowski et al. 2008). Because > 99% of lead in blood is present in red blood cells, lack of adjustment for a measure of red blood cell concentration in a population with anemia has the potential to influence blood lead level measurements (deSilva 1984; Kim and Lee 2012; Skerfving and Bergdahl 2007). This could introduce an exposure measurement error, particularly in populations with CKD, given the common comorbidity of secondary anemia and wide variation in hemoglobin level. However, the relevance of measurement error is unclear, given the finding in our study that the association between lead and GFR is strongest among those without anemia, with or without hemoglobin adjustment. Our finding raises potential questions about the role and timing of lead in the pathogenesis of CKD, if it is indeed pathogenic at these levels. It is acknowledged that this is a post hoc analysis and these questions are not easily addressable with a cross-sectional analysis, but the examination of the impact of hemoglobin level/anemia status on blood lead levels and CKD progression deserves further study.

Although the mechanisms of lead nephrotoxicity are incompletely characterized, and no studies in humans have identified a mechanism for direct nephrotoxicity at blood lead concentrations < 10 µg/dL, evidence primarily from animal and *in vitro* studies has elucidated multiple cellular and molecular mechanisms showing that lead exposure results in oxidative stress and inflammation. Chronic lead exposure results in decreased nitric oxide and impaired nitric oxide signaling, alterations in vasoactive prostaglandins, alterations in the renin–angiotensin system, and alteration of multiple molecules involved in endothelial and vascular function *in vitro* and *in vivo* in rats (Rodriguez-Iturbe et al. 2005; Vaziri 2008). Through such mechanisms, either directly or indirectly via increases in blood pressure and arteriosclerosis, chronic lead exposure may affect the nephron and thus kidney function.

This study has limitations. Although blood lead concentration is the most commonly used biomarker to estimate total lead body burden in research studies and the clinical arena primarily due to feasibility, it has known limitations. Because of the short half-life of blood lead (approximately 30 days), it not possible to know whether the level of blood lead is attributable to acute or chronic exposure, or rather is reflecting the slow elimination kinetics of lead in bone, the main reservoir of lead in the body (ATSDR 2007). Thus, a single blood lead level may not accurately portray either the duration or degree of exposure (Hu et al. 1998). Additionally, the impact of factors such as bone disease and inflammation that may be prevalent in advanced CKD on the bioavailability of lead, and thus blood lead levels, is poorly described (Evans and Elinder 2011). The cross-sectional design of this study does not allow for the determination of causality, including the possibility that lead levels rise as a consequence of lower GFR (“reverse causality”). Given that all children in this study had kidney disease, the role of kidney disease on the pattern of results is not certain. Finally, a number of sensitivity and post hoc analyses were included in this study to examine the consistency of the results. Because *a priori* rationales did not exist for some sensitivity analyses, results should be interpreted with caution.

In contrast to most studies examining the association of lead levels with GFR, our study benefited from direct GFR measurements. Despite the limited precision and accuracy of GFR estimating equations, our study found similar results using measured GFR and the bedside CKiD GFR estimating equation which incorporates creatinine and height (Schwartz et al. 2009), supporting the use of creatinine-based estimating equations in other studies.

## Conclusion

This study of a relatively large cohort of children with CKD and blood lead levels representative of current environmental levels of exposure did not find a significant association between lead and directly measured GFR. A negative association between lead and GFR was observed among children with CKD caused by glomerular disease, and among children who were not anemic. These findings, including the impact of anemia/hemoglobin adjustment on blood lead levels and associated outcomes, particularly in populations with CKD, deserve further investigation.
